# Reviewed article: Carvalho, SMO; Andrade, JX; Nascimento, FF; Rodrigues, MTP; Mascarenhas, MDM. Temporal trends in acquired syphilis detection, hospitalizations, and mortality: a time series analysis, Brazil, 2011-2023. Epidemiol Serv Saude. 2026:35;e20251388

**DOI:** 10.1590/S2237-96222026v35e20251388.a

**Published:** 2026-11-02

**Authors:** Junio Eduvirgem

**Affiliations:** 1 Universidade Federal da Grande Dourados, Dourados, MS, Brazil Universidade Federal da Grande Dourados Dourados MS Brazil

## Peer review A

### Questionnaire

Does the manuscript contain new and significant information to justify publication? Yes 

Does the Abstract (Summary) clearly and accurately describe the content of the article? Yes 

Is the problem significant and concisely stated? Yes 

Are the methods described comprehensively? Yes 

Are the interpretations and conclusions justified by the results? Yes 

Is adequate reference made to other work in the field? Yes 

### Manuscript Structure:

Length of article is: Adequate

Number of tables is: Adequate

Number of figures is: Adequate

### Rating

Interest: Excellent

Quality: Excellent

Originality: Excellent

Overall: Excellent

Recommendation: Major Revision 

### Comments

1. Sugiro análise estatística adicional por terem empregado uma abordagem que, embora válida para correção de autocorrelação (Prais-Winsten), pode ser limitada para captar mudanças de tendência estrutural (pontos de inflexão). Recomendo que os autores avaliem a aplicação de Modelos de Regressão por Pontos de Inflexão (Joinpoint Regression), que permitiriam identificar se o crescimento da sífilis foi constante ou se houve períodos de aceleração/desaceleração mais precisos, oferecendo uma interpretação epidemiológica mais robusta para o planejamento de serviços de saúde.
2. Os autores não detalham quais códigos CID-10 foram utilizados para filtrar as internações e óbitos, o que dificulta a reprodutibilidade e pode gerar viés de seleção. Sugiro esclarecimento quanto ao apontamento para melhor reprodutividade dos dados.
3. A discussão sobre o impacto da pandemia na modelagem estatística e a definição precisa dos critérios de busca no SIH/SIM precisam de maior clareza para garantir a robustez das conclusões apresentadas.
4. As Tabelas 1, 2 e 3 precisam ser revisadas quanto a formatação para garantir clareza, precisão e evitar redundância com o texto.
5. A Figura 1 precisa ser revisada quanto a formatação (letras de eixos X e Y em fonte maiores) e melhor identificação de cada gráfico (A-1; A-2, respectivamente) para garantir clareza, precisão e evitar redundância com o texto. Sugiro readequar a descrição da legenda da Figura 1.

